# CRISPR–Cas9 Genetic Analysis of Virus–Host Interactions

**DOI:** 10.3390/v10020055

**Published:** 2018-01-30

**Authors:** Makda Gebre, Jason L. Nomburg, Benjamin E. Gewurz

**Affiliations:** 1 Harvard Program in Virology, Harvard Medical School, Boston, MA 02115, USA; Makda_Gebre@g.harvard.edu (M.G.); jnomburg@g.harvard.edu (J.L.N.); 2 Division of Infectious Disease, Department of Medicine, Brigham & Women’s Hospital, Boston, MA 02115, USA; 3 Department of Microbiology and Immunobiology, Harvard Medical School, Boston, MA 02115, USA; 4 Broad Institute of Harvard and Massachusetts Institute of Technology, Cambridge, MA 02142, USA

**Keywords:** genome engineering, CRISPR screen, Cas9, Epstein–Barr virus, host dependency factor, CRISPR interference, CRISPR activation, combinatorial CRISPR

## Abstract

Clustered regularly interspaced short palindromic repeats (CRISPR) has greatly expanded the ability to genetically probe virus–host interactions. CRISPR systems enable focused or systematic, genomewide studies of nearly all aspects of a virus lifecycle. Combined with its relative ease of use and high reproducibility, CRISPR is becoming an essential tool in studies of the host factors important for viral pathogenesis. Here, we review the use of CRISPR–Cas9 for the loss-of-function analysis of host dependency factors. We focus on the use of CRISPR-pooled screens for the systematic identification of host dependency factors, particularly in Epstein–Barr virus-transformed B cells. We also discuss the use of CRISPR interference (CRISPRi) and gain-of-function CRISPR activation (CRISPRa) approaches to probe virus–host interactions. Finally, we comment on the future directions enabled by combinatorial CRISPR screens.

## 1. Introduction to CRISPR

**Figure 1:**
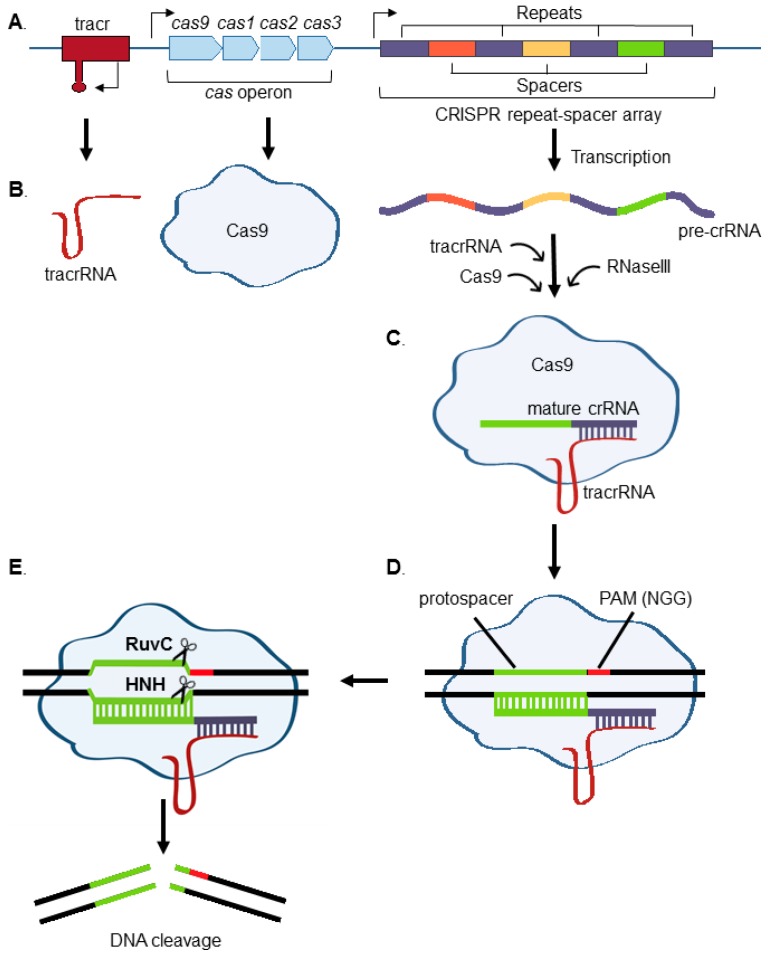
Type II CRISPR–Cas9 overview. (**A**) Key components of the *Streptococcus pyogenes* CRISPR–Cas system are shown. These include the trans-activating crRNA (tracr) locus, *cas* gene operon, and CRISPR repeat-spacer array; (**B**) the CRISPR repeat-spacer array is transcribed into pre-crRNA, which includes archived spacer sequence derived from foreign DNA. The pre-crRNA transcript is cleaved and processed into a single spacer-repeat sequence by a complex of RNaseIII, tracrRNA, and Cas9; (**C**,**D**) Cas9 forms a complex with the tracrRNA and mature crRNA complex, which then uses the protospacer RNA-targeting sequence to bind DNA with complementary sequence; (**E**) Cas9 RuvC and HNH nuclease domains create a double-strand break in the crRNA-paired sequence, so long as there is a protospacer adjacent motif (PAM) immediately after the targeting sequence. For the commonly used *S. pyogenes* Cas9, the PAM sequence is NGG, where N can be any nucleotide.

Clustered regularly interspaced short palindromic repeats (CRISPR)–Cas are the RNA and protein-based adaptive immune system that protect bacteria and archaea against invading viruses and foreign nucleic acids [1,2]. Bacterial CRISPR loci are comprised of a Cas operon and a repeat-spacer array. CRISPR spacers are archived sequences, typically 23–44 base pairs in length, copied from phage or plasmid DNA that previously invaded the bacterium [3]. These sequences are interspersed between repeat sequences and are located adjacent to the CRISPR-associated (Cas) genes, which encode Cas ribonucleases (Figure 1). The acquisition of spacers into the prokaryotic genome allows for the recognition and destruction of nucleic acid matching the archived sequence upon subsequent invasion.

The CRISPR locus is transcribed into pre-CRISPR RNA (crRNA), which is then processed by Cas proteins and accessory factors into mature crRNA [4]. Cas proteins are programmed by crRNA to cut nucleic acid flanked by a short protospacer adjacent motif (PAM) sequence [5]. There are three distinct CRISPR–Cas systems, which differ in how they process pre-crRNA. Type II systems are the most widely used for eukaryotic genome engineering, in large part because a single protein, Cas9, is required to locate and cleave the target sequences. Trans-activating crRNA (tracrRNA) base-pairs with crRNA transcript repeat regions. Together, crRNA and tracrRNA are sufficient to guide Cas9 to the target sites and to activate its nuclease activity against DNA complementary to the crRNA [4]. TracrRNA and crRNA are typically fused into a single-guide RNA (sgRNA) transcript for eukaryotic genome engineering. The Cas9 HNH domain cleaves the DNA strand complementary to the guide RNA, while the RuvC domain cleaves the noncomplementary strand (Figure 1) [6,7].

*Streptococcus pyogenes* Cas9 (SpCas9) is the most widely used for eukaryotic genome engineering. However, a drawback is its large ~4.2 kilobase size, which reduces lentivirus yield. Consequently, a common approach for high throughput approaches is to first establish cell lines that stably express SpCas9 and to then transduce the cells with a lentivirus sgRNA library [8,9]. Alternatively, SpCas9 expression can be inducible or transient [10,11], or smaller bacterial *cas9* genes can be used. For instance, *Staphylococcus aureus cas9* (SaCas9) is ~3.2 kilobases and uses the PAM sequence NNGRRV, where R is either A or G and V is either A, C, or G [12,13,14]. Thus, the use of SaCas9 also enables the targeting of sites with these PAM sequences, distinct from the SpCas9 NGG PAM sequence*.* Molecular evolution approaches have been used to further expand the range of PAM sequences that can be targeted by SaCas9 [15]. Lentiviral sgRNA libraries are also starting to become available for SaCas9 [12]. For high-throughput genetic screens, replication-defective lentiviral vectors are used to deliver sgRNA and a selectable marker to Cas9+ target cells [16,17]. Custom sgRNAs contain 20 base pairs of crRNA sequence complementary to the target DNA site [9,17].

## 2. Use of Nonhomologous End-Joining (NHEJ) to Generate Functional Knockouts

Mammalian genomic double-stranded breaks (DSB) are predominantly repaired by the nonhomologous end-joining (NHEJ) pathway. Briefly, the ku70–ku80 heterodimer binds DSBs and recruits additional factors, including the DNA-dependent protein kinase catalytic subunit (DNA-PKcs) and the endonuclease Artemis [18,19]. DNA-PKcs phosphorylates Artemis, which produces a blunt 5′ end and a 4–5 nucleotide 3′ overhang [20]. A DNA polymerase fills in the overhangs to make them compatible for ligation [21]. Thus, NHEJ is error-prone and frequently introduces frameshift insertions, deletions, and/or stop codon mutations. When targeted to coding regions, NHEJ frequently disrupts the expression of the encoded protein [22,23,24,25,26]. sgRNAs targeting early exons therefore result in functional knockouts, which are frequently biallelic. CRISPR-induced DSB and NHEJ have been used to introduce indels into DNA virus genomes, including those of adenovirus, herpes simplex virus, hepatitis B virus, human papillomavirus, JC virus, and Epstein–Barr virus [27,28]. Interestingly, CRISPR editing could even be achieved with vaccinia virus, which replicates its DNA in the cytosol [29].

## 3. Introduction to CRISPR-Pooled Screens for Host–Virus Studies

Pooled screens are an economical and rapid way to test the effects of large numbers of CRISPR genetic perturbations on a phenotype. In contrast to arrayed screens, in which a single sgRNA is tested in each well, CRISPR-pooled screens use lentivirus libraries to deliver large numbers of sgRNAs to a cell population, in order to create a gene-edited library. CRISPR has the advantage over RNAi of producing homozygous null phenotypes, which can allow for greater phenotypic penetrance. A limitation of CRISPR knockout screens is that essential genes can easily be missed, since their knockout causes cell death or proliferation arrest, resulting in loss from the library. Nonetheless, CRISPR–Cas9-pooled screens have enormous potential for the discovery of host factors critical for viral pathogenesis and promise to highlight novel therapeutic targets.

**Figure 2:**
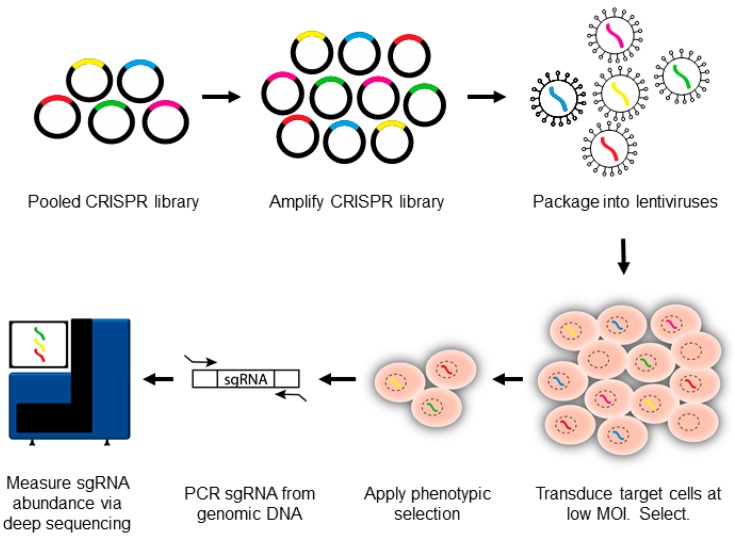
CRISPR–Cas9-pooled screen overview. Single-guide RNA (sgRNA) libraries are initially synthesized as lentiviral vectors and are then amplified and packaged into lentiviruses. The lentiviral vector encodes an sgRNA and a selectable marker, typically puromycin. Several libraries are now commercially available. Lentivirus pools are then used to transduce Cas9+ target cells at low multiplicity of infection (MOI). A low MOI is used to avoid coinfection by multiple lentiviruses. A positive or negative selection pressure is used to identify cells with a desired phenotype. The genomic DNA is extracted from input versus selected cell pools, and the integrated sgRNA sequences are PCR-amplified. The sgRNA abundance is then determined by next-generation DNA sequencing.

CRISPR-pooled screen cells typically begin by transducing *S. pyogenes* Cas9+ target cells with a lentiviral sgRNA library (Figure 2). Multiple human or mouse genomewide sgRNA libraries are now commercially available [9,24,30,31,32]. sgRNA design algorithms are rapidly improving [8], and Cas9 off-target effects are already considerably less than those observed with RNA interference [13,33,34,35,36]. Nonetheless, some sgRNAs target large numbers of genes, and off-target effects remain an important source of false-positive results. To overcome this barrier, sgRNA libraries include 4–10 sgRNAs against each gene of interest [9,24,30,31,32]. This ‘reagent-redundancy’ approach [37] enables the identification of high-confidence hits, for which independent sgRNAs produce the same phenotype. Since each sgRNA has a distinct off-target signature, the concordant phenotype likely results from on-target activity. For human genomewide screens, this results in large numbers (~10^5^) of sgRNAs to be tested [9,38,39,40].

Lentiviral transduction is performed at a low multiplicity of infection (MOI), typically 0.3, to avoid coinfection by multiple viruses [9]. The transduced cells are selected, often by puromycin. The library is then allowed to grow for a short period of time to permit phenotypic maturation, typically 7–14 days. This prescreen phase allows for decay of the target protein, synthesized prior to CRISPR gene editing. The CRISPR knockout library is then tested by a phenotypic screen with the virus or the viral protein of interest.

Positive or negative selection approaches have successfully been used with CRISPR-pooled screens. For positive selection screens, gene-edited cells with a desired phenotype are obtained, for instance by cell survival selection, fluorescence-activated cell sorting, or antibody-based bead selection. For negative selection screens, gene-edited cells that have dropped out of a population are identified, for example because the sgRNA causes decreased proliferation, cell death, or loss of a protein marker. This can include synthetic interactions with a viral protein or with a virus-induced host gene. Integrated sgRNA sequences serve as convenient barcodes. The PCR-amplified sgRNA abundances in the input (preselection) library versus the selected population are then quantitated by deep sequencing, using multiple screen replicates (Figure 2) [41]. Adequate target cell numbers are required to ensure robustness in sgRNA sampling [9]. We typically use target cell libraries with an average of 500 cells per sgRNA, resulting in ~40 million cells for a library with 4 sgRNAs against each human gene [9].

Algorithms such as STARS or MAGeCK are used to identify statistically significant hits from the deep sequencing data [8,9,38,42], typically by comparing input versus selected cell populations. STARS and MAGeCK identify robust hits, for which multiple sgRNA abundances differ significantly between input and selected populations. The use of a stringent multiple hypothesis-adjusted p-value cutoff, such as a false discovery rate or *q*-value < 0.05, can reduce false positive signals arising from large numbers of tests. For hit validation, we typically begin by confirming the screen phenotype with focused studies of two independent sgRNAs per screen hit of interest. In selected cases, hits can then be further validated by cDNA rescue. cDNA with silent PAM site mutations, which are sufficient to abrogate Cas9 cutting, are stably expressed. For SpCas9, either guanine in the “NGG” PAM motif can be mutated to any other DNA base. Assays are then performed to determine whether this cDNA rescues phenotypes caused by the editing of the endogenous target gene.

## 4. Pooled Screens for RNA Virus Host Dependency Factors

CRISPR-pooled screens have successfully identified host factors critical for RNA virus entry, replication, and spread. These screens used the rescue from viral cytopathic effect as an endpoint, where CRISPR knockouts enabled cells to survive an otherwise lethal viral challenge. The loss of identified host factors blocked key aspects of viral replication and thereby reduced cytotoxicity. Excellent recent reviews describe these analyses [43,44], which will therefore only be covered here briefly. Collectively, the studies outlined below, which we believe to be a comprehensive list of CRISPR-pooled screens of RNA virus biology at the time of this publication, highlight the significant potential for CRISPR loss-of-function analysis for studies of host dependency factors.

CRISPR–Cas9 screens independently identified endoplasmic reticulum (ER) pathways important for flavivirus replication. These include screens of West Nile [31,45], dengue [44], Zika [46], and hepatitis C virus [47] replication, which used the rescue from viral cytopathic effect as the method of positive selection. The N-linked glycosylation pathway, ER-associated degradation, and ER signal peptide pathway strongly scored in these flavivirus replication screens. Encouragingly, a significant hit overlap was observed between these screens by independent laboratories. Furthermore, a significant percentage of hits were functionally interconnected, often by protein–protein interactions. For instance, in a dengue replication screen, four ER translocon components, seven ER-associated degradation pathway components, and seven oligosaccharyl-transferase factors were amongst the top 40 hits [47]. Further increasing the confidence in these CRISPR results, these same pathways were identified by haploid cell transposon insertion mutagenesis genetic screens, and multiple hits were validated by cDNA rescue.

CRISPR-pooled screens have already revealed several viral receptors. Independent screens identified CD300LF as the receptor for murine norovirus (MNV) infection [48,49]. CD300 Antigen-Like Family Member F (CD300LF) expression was sufficient to confer human cell susceptibility to MNV infection, and CD300LF knockout protected cell lines and mice from MNV [48,49]. Likewise, CRISPR was used to screen for host factors important for human rhinovirus (HRV) infection. Intracellular adhesion molecule 1 (ICAM1), the known HRV receptor, was one of the two high-confidence hits. The exocyst-targeting and vesicular transport pathway component Exocyst Complex Component 4 (EXOC4) was the other hit, whose role in supporting HRV infection remains to be further characterized [44]. A CRISPR–Cas9 screen for T cell factors important for C-C motif chemokine receptor 5 (CCR5)-tropic human immunodeficiency virus (HIV) infection identified the obligatory receptors CD4 and CCR5. It also found the protein sulfation pathway components Solute Carrier Family 35 Member B2 (SLC35B2) and Tyrosylprotein Sulfotransferase 2 (TPST2) as critical for HIV entry [50]. TPST2 mediates sulfation of CCR5 N-terminal residues important for the interaction with HIV gp120 [51]. Interestingly, Activated Leukocyte Cell Adhesion Molecule (ALCAM), which drives homotypic interactions and is expressed by activated T cells, was also found to be critical for HIV spread to neighboring T cells.

A limitation evident from these elegant CRISPR-pooled screens, as well as from related haploid cell screens [43,44], is that the hits can be biased towards factors important for viral entry. Potential strategies for increasing the sensitivity for other types of host dependency factors include the use of milder selection approaches and shorter screen periods. For instance, a flow cytometry readout enabled the identification of a broad array of cell factors important for primary mouse dendritic cell response to lipopolysaccharide [52].

## 5. Genomewide Screen for EBV-Transformed B Cell Host Dependency Factors

Epstein–Barr virus (EBV) causes ~200,000 human cancers annually [53]. EBV is associated with multiple human malignancies, including immunoblastic lymphomas of immunosuppressed hosts, Burkitt lymphoma (BL), Hodgkin lymphoma, T- and NK-cell lymphomas, nasopharyngeal and gastric carcinomas [54,55,56,57,58,59]. EBV efficiently transforms primary human B cells into immortalized lymphoblastoid cell lines (LCL), which are models for EBV-driven immunosuppression-associated lymphomas. EBV also causes endemic Burkitt lymphoma [54,56,60].

CRISPR is a powerful tool for genetic analysis in EBV-transformed cells. For unclear reasons, Cas9 activity can vary significantly between cell lines and does not necessarily correlate with its expression level [61]. We have also observed this phenomenon in EBV+ B-cell lines, where the frequency of Cas9-induced indels appears to vary significantly across cell lines. To identify LCLs and BLs with robust CRISPR activity following stable Cas9 expression, we used a convenient green fluorescence protein reporter assay [9,41,62,63]. We did not observe toxicity related to stable Cas9 expression in LCLs or BLs. Established Cas9+ cell lines maintain its expression for months in the absence of antibiotic selection.

In early CRISPR studies with BL cells, coexpression of sgRNAs targeting multiple EBV episome sites caused viral genome loss and proliferation arrest [64,65]. CRISPR was also used to mutate EBV genomes in gastric carcinoma and nasopharyngeal carcinoma cells [66,67]. In early LCL Cas9 studies, CRISPR was used to test host dependency factor roles of the EBNA3-associated Ubiquitin Specific Protease 46 (USP46) in cell growth [68] and of the EBV-activated linear ubiquitin assembly complex subunit HOIP in LCL growth and survival [69]. More recently, CRISPR was used to study CD63 roles in Latent Membrane Protein 1 (LMP1) exosomal packaging, ephrin receptor A2 role in EBV entry into epithelial cells [70,71], and the roles of IL-1 Receptor Associated Kinase 4 (IRAK4) [72] or B-cell Lymphoma 6 (BCL6) [73] in the suppression of lytic replication.

EBV+ BL and immune-suppression lymphomas arise by distinct oncogenic pathways, express different EBV latency programs, and are treated by distinct therapeutic regimens. EBV+ BL cells express the viral latency I program, comprised of the Epstein–Barr nuclear antigen (EBNA) 1 and non-coding RNAs. LCLs express the EBV latency III program, including six EBNA, three latent membrane proteins, and non-coding RNAs. EBV latency factors lack enzymatic activity and have not yet proven to be druggable. Downstream host dependency factors are therefore potential therapeutic targets.

**Figure 3:**
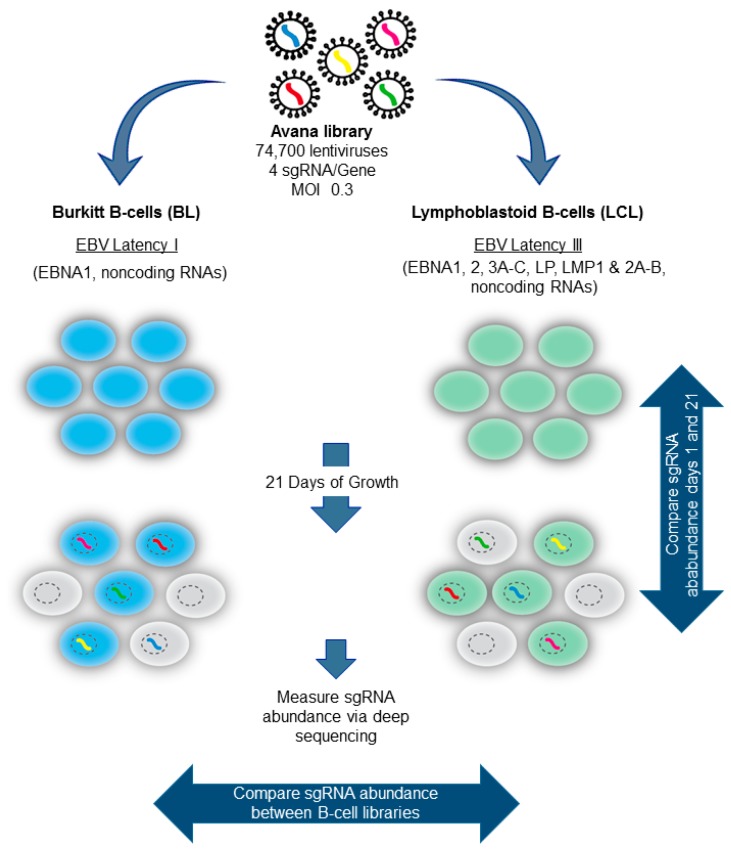
CRISPR-pooled screen for EBV-Transformed B cell host dependency factors. EBV+ P3HR1 Burkitt lymphoma (BL) or GM12878 lymphoblastoid cell lines (LCLs) that stably express SpCas9 were transduced with the lentivirus Avana sgRNA library at a MOI of 0.3. The transduced cells were puromycin-selected, and then passaged for 21 days. Library input and day 21 sgRNA abundances were quantitated by deep-sequencing. The STARS algorithm was used to identify sgRNAs that were significantly enriched or depleted in day 21 cell pools vs. input, or between day 21 P3HR1 and GM12878 pools.

To systematically identify host dependency factors important for BL versus LCL growth and survival, we performed genomewide CRISPR–Cas9 loss-of-function pooled screens [74]. Cas9+ LCL and BL cell lines were established, and clones with robust Cas9 activity were identified by use of the lentiviral pXPR-011 GFP reporter system [41]. BL and LCL CRISPR–Cas9 gene edited libraries were then constructed by infecting ~130 million cells of each cell line with the Avana lentivirus sgRNA library at a MOI of 0.3, to minimize lentivirus coinfection (Figure 3). The Avana library is comprised of lentiviruses that express one sgRNA, each of which targets a human gene. Each human gene is targeted by four independent Avana library sgRNAs, on average [8]. The transduced cells were selected by puromycin resistance. Four biological replicate libraries were established for each cell line. Following puromycin selection, the cells were passaged for 21 days to provide a strong selective pressure for sgRNAs that enhanced or suppressed cell growth or survival.

**Figure 4:**
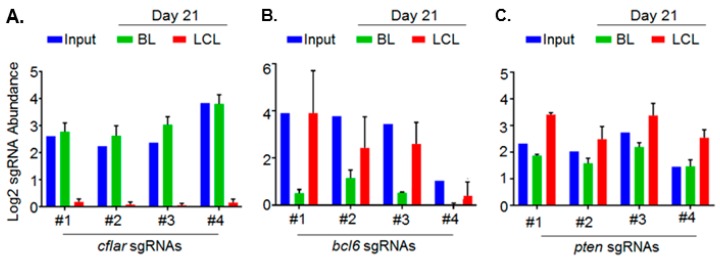
Representative Epstein–Barr (EBV)-transformed B cell CRISPR-pooled screen hits. (**A**) Log2 abundances of the four Avana library sgRNAs that target CASP8 And FADD Like Apoptosis Regulator (*cflar*), which encodes Cellular FLICE-Like Inhibitory Protein (cFLIP), in the indicated populations. All four *cflar* targeting sgRNAs are markedly depleted from the day 21 LCL pool, but not from the day 21 BL cell pool, relative to input levels, suggesting a key cFLIP role as an LCL-selective dependency factor; (**B**) all four Avana sgRNAs targeting B-cell lymphoma 6 (*bcl6*) are depleted from the day 21 BL pool and to a significantly lower extent from the day 21 LCL pool, indicating a BL-selective dependency factor role; (**C**) all four sgRNAs against the Phosphatase And Tensin Homolog (*pten*) are enriched compared to input levels in the day 21 LCL pool, suggesting that PTEN loss enhances LCL growth and/or survival.

As described above, integrated sgRNA sequences serve as bar codes. Input and day 21 sgRNA abundances were quantitated by next-generation sequencing of PCR-amplified sequences. The STARS algorithm was then used to identify high-confidence hits. To highlight the dependencies of each EBV latency state and to filter essential B cell housekeeping genes, we focused on hits with significant differences in sgRNA abundance in day 21 BL versus LCL libraries (Figure 3). Using a multiple hypothesis test-adjusted *q* < 0.05 cutoff, 87 host dependence factors were found to be selectively important for LCL growth and survival versus 57 for BL [74]. In support of the CRISPR screen results, the hits included well-characterized LCL and BL dependency factors. For instance, a top BL-selective hit was the germinal center transcription repressor BCL6, which has important oncogenic roles in BL pathogenesis [75,76] (Figure 4). Interestingly, despite this key BCL6 role in BLs, EBNA3C was recently found to target BCL6 for degradation by the ubiquitin-proteasome pathway in LCLs [77]. Indeed, we found that sgRNAs-targeting BCL6 were significantly less depleted from the day 21 LCL library (Figure 4). Likewise, top LCL-selective hits included genes previously defined to be critical for LCL growth and survival. These included the genes encoding LMP1–NF-κB pathway components IκB kinase β (IKKβ), HOIL-1-Interacting Protein (HOIP), and p52 [78,79,80,81,82,83], the EBNA cofactor Recombination Signal Binding Protein For Immunoglobulin κ J Region (RBP-Jκ) [84,85,86,87,88,89,90], and the EBNA3-associated proteins WD Repeat Domain 48 (WDR48) and C-Terminal Binding Protein 1 (CTBP1) [68,91,92].

Consistent with the on-target effects underlying our high-confidence hits, at least two, and usually three of the four sgRNAs per hit scored independently. Screen biological quadruplicate replicates were highly concordant. Genes identified as key BL and LCL dependency factors are expressed in these cell types, and LCL-selective hits were highly enriched for EBV latency III program-induced host genes. Notably, the 87 LCL-selective hits were significantly enriched for EBV-upregulated host genes. By contrast, none of the >1000 host genes suppressed by >1.5 fold upon primary B-cell EBV infection scored as LCL dependency factors [93]. Interestingly, the screen also identified putative tumor suppressors. For instance, all four sgRNAs against the gene encoding Phosphatase and Tensin Homolog (PTEN), which opposes Phosphoinositide-3-Kinase (PI3K), were significantly enriched after 21 days of growth (Figure 4). This result suggests that although an EBV miRNA targets PTEN [94], residual PTEN nonetheless fine-tunes LCL PI3K activity.

We confirmed several top hits by expression of sgRNA-resistant cDNA with silent PAM site mutations. The remainder of this section illustrates how we followed several top LCL screen hits to provide an example of downstream studies arising from a CRISPR-pooled screen for host dependency factors.

The top LCL-selective screen hit was *cflar*, which encodes CASP8 And FADD Like Apoptosis Regulator (cFLIP), a potent antagonist of apoptosis, necroptosis, and autophagy pathways. cFLIP is highly induced in LCLs by the LMP1–NF-κB pathway [81,95,96] and by an EBV super-enhancer that loops to the cFLIP promoter [79,97,98]. However, its role as an LCL dependency factor had not previously been characterized. cFLIP knockout caused rapid caspase activation and LCL death. Yet, cFLIP is not expressed in many EBV+ BL or in primary B cells. How then does EBV latency III infection make cFLIP a critical dependency factor? To address this question, we next used CRISPR to test whether LCL death receptor activation creates the role of cFLIP as a dependency factor. CRISPR knockout of the type I Tumor Necrosis Factor (TNF) receptor (TNFR1), but not of the related Fas death receptor, rescued LCLs from cFLIP loss [74]. These results suggest that TNFα production, in response to EBV latency III infection, creates the synthetic requirement for cFLIP to block TNFR1-driven programed cell death. Furthermore, we could rescue the endogenous cFLIP knockout by the expression of just the cFLIP isoform which blocks death receptor-induced apoptosis, but not the necroptosis pathway. This result is consistent with a model in which the key cFLIP dependency factor role is to block the TNFα-driven extrinsic apoptosis pathway.

The EBV latency III-induced transcription factors Basic Leucine Zipper ATF-Like Transcription Factor (BATF) and Interferon Regulatory Factor 4 (IRF4) were also identified as top LCL-selective dependency factors. BATF-containing AP-1 transcription factors and IRF4 cooperatively bind to composite AP-1-ISRE DNA motifs. IRF4 also associates with EBNA3C [99], and a BATF–IRF4 DNA motif is enriched at EBNA3 and 3C binding sites [100,101]. Furthermore, IRF4 is targeted by an EBV super-enhancer that loops to its promoter from a long distance upstream, also suggesting a key LCL IRF4 role [98]. To gain further insights into key LCL IRF4 and BATF dependency factor roles, we performed RNA deep sequencing (RNAseq) in IRF4 or BATF gene-edited LCLs, prior to the onset of apoptosis. The tumor suppressor *bcl2l11*, which encodes B-cell Lymphoma 2 (BCL2) Interacting Mediator of Cell Death (BIM), a proapoptosis factor that blocks BCL2 family members, was found to be amongst the most highly induced LCL genes upon knockout of either IRF4 or BATF. EBNA3A and 3C suppress BIM expression [102,103,104,105] and co-occupy a site upstream of BIM. Using ENCODE ChIP-seq from the same LCL used for our screen [74], we observed IRF4 and BATF co-occupancy with EBNA3 at this site. These data are consistent with a model where EBV-induced BATF and IRF4 bind to a composite DNA site to recruit EBNA3A/C and polycomb repressor complexes that include the LCL dependency factor CTBP1. Notably, BIM knockout was not sufficient to rescue LCLs from subsequent IRF4 or BATF loss, suggesting additional key BATF and IRF4 dependency factor roles in LCL survival. Our RNAseq data suggest potential key roles in Myelocytomatosis Viral Oncogene Homolog (MYC*)* expression, and in suppression of interferon responses, as areas for future investigation.

The related transcription factor Interferon Regulatory Factor 2 (IRF2) scored as a top LCL-selective dependency factor. RNAseq analysis of IRF2-knockout LCLs uncovered the intriguing finding that IRF2 and IRF4 frequently oppose one another in LCLs, yet they are each are key EBV-induced LCL dependency factors [74]. To further characterize this finding, we performed GO analysis on control versus IRF2-KO LCLs, which revealed *myc*, and MYC target genes, as downstream IRF2 LCL targets. Notably, the tumor suppressor B-Lymphocyte-Induced Maturation Protein 1 (Blimp1) (encoded by *prdm1*) was 10-fold induced by IRF2 LCL knockout, and Blimp1 is a potent *myc* repressor [74]. In support of a key IRF2 role in LCL Blimp1 suppression, Blimp1 knockout partially rescued LCLs from IRF2 knockout-induced programmed cell death.

EBV has also evolved an EBNA3A/3C-dependent epigenetic mechanism to suppress Blimp1 [106]. Intriguingly, primary B cell infection by EBV lacking the EBNA3 locus results in failure to suppress Blimp1 and the outgrowth of plasmablast, rather than lymphoblastoid cells [106]. Yet, coexpression of LMP1 and LMP2A in germinal center B cells upregulate IRF4 and Blimp1 and results in plasmablast differentiation in a mouse model [107]. Taken together, our results suggest that IRF2, EBNA3A, and 3C may jointly repress Blimp1 expression in the context of EBV-driven IRF4 expression. IRF2 may recruit EBNA3 proteins and corepressors to the *prdm1* locus. Alternatively, IRF2 may recruit cellular repressors independently from EBNA3 or it may counteract IRF4 effects at *prdm1* to suppress Blimp1 expression and plasmablast differentiation. A second dependency-factor role for Blimp1 suppression is to prevent the induction of EBV lytic replication [108,109,110,111]. The EBV miRNA BHRF1-2 further suppresses its expression [108]. Taken together, these studies indicate that EBV has evolved clever strategies to induce IRF4-dependent growth and survival and to then suppress the expression of its downstream tumor suppressor target Blimp1.

In summary, CRISPR-pooled screens identified high-confidence dependency factors necessary for EBV-transformed B-cell growth, including multiple druggable targets for which antagonists are in clinical use for other oncology indications. Limitations of this CRISPR-pooled screen approach for EBV-induced host dependency factors include the potential for false-negative results, for example caused by redundancy between host dependency factors, instances where sgRNAs fail to induce functional knockout of host genes, or the requirement for in vivo microenvironments. Interestingly, CRISPR loss-of-function pooled screens have recently been performed in vivo using mutagenized murine cancer cell lines. These studies revealed cell intrinsic suppressors of non-small-cell lung cancer growth and metastasis [112] and novel melanoma cell intrinsic targets that synergize with or cause resistance to PD-1 immune checkpoint blockade [113]. Thus, CRISPR-pooled screens using xenograft and perhaps also humanized mouse models may likewise identify additional EBV-driven host dependency factors important for growth, survival, or response to chemical antagonists in the context of physiologic microenvironments. It will also be interesting to test how early after primary human B cell infection the identified dependency factors become required for EBV-driven transformed cell growth and survival, and whether targeting these host factors during high-risk periods can prevent the outgrowth of EBV-driven lymphoproliferative diseases in highly immunosuppressed patients.

## 6. CRISPR Interference and CRISPR Activation

CRISPR interference (CRISPRi) and CRISPR activation (CRISPRa) are genetic perturbation techniques that utilize modified Cas9 variants as transcription factors, rather than as endonucleases. Point mutations in the Cas9 endonuclease cleavage domains abrogate DNA cleavage by dead Cas9 (dCas9) [114]. dCas9 is nonetheless programmed by sgRNAs to occupy specific genomic sites, which can then inhibit RNA polymerase II (Pol II) elongation [114]. The great strength of this system, however, is the ability to modify dCas9 with various activating or inhibitory elements. dCas9 can be fused with transcription inhibitory domains to more strongly block target gene transcription (CRISPRi), or with transcription activation domains to activate transcription (CRISPRa).

**Figure 5:**
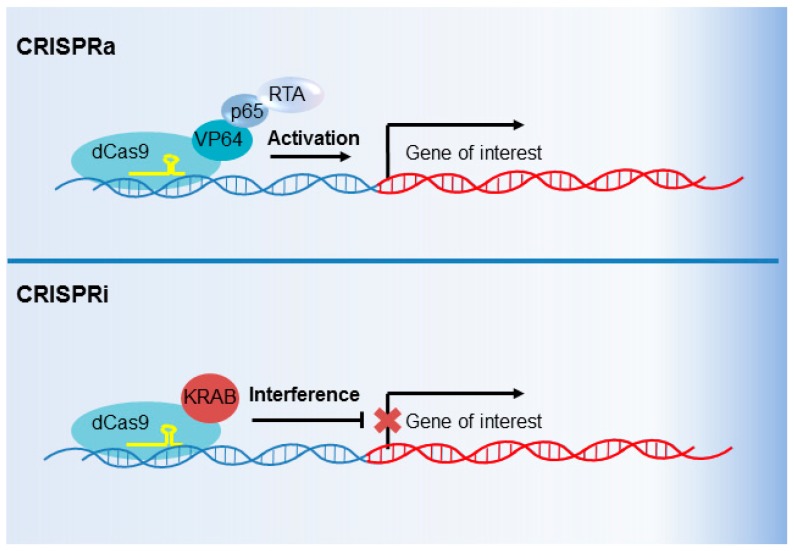
CRISPR activation (CRISPRa) and CRISPR interference (CRISPRi) overview. Catalytically inactive “dead” Cas9 (dCas9), in which point mutations abrogate DNA cleavage by the HNH and RuvC domains, can be repurposed as a transcription activator (CRISPRa) or repressor (CRISPRi). For CRISPRa (**top**), dCas9 is fused to a transcription activator. An sgRNA (yellow) programs dCas9 fusion to activate the transcription of the targeted promoter. A commonly used CRISPRa system harnesses four herpes simplex virus VP16 molecules (so called VP64), the NF-κB transcription factor p65, and the EBV immediate-early transcription activator RTA. For CRISPRi (**bottom**), dCas9 is fused to a transcription repressor. A common CRISPRi system uses the Krüppel-associated box (KRAB) domain. dCas9–KRAB directs heterochromatin formation at the promoter targeted by the sgRNA, reducing target gene transcription.

CRISPRi can utilize any of a number of transcription inhibitory domains to repress transcription [9,115]. For example, a fusion of dCas9 and the Krüppel-associated box (KRAB) domain strongly decreases transcription of a sgRNA-specified target gene by promoting the formation of heterochromatin at its promoter [114,116,117] (Figure 5). In contrast, CRISPRa functions through the fusion of dCas9 with domains that instead activate transcription. One or multiple transcription activating domains can be fused to dCas9 [115,118,119,120]. For instance, fusion of dCas9 with herpes simplex virus VP16 is frequently used to upregulate target gene expression [116,121] (Figure 5). Furthermore, resources have been established for the design and implementation of focused or genomewide CRISPRi or CRISPRa analyses [122,123]. Of note, Cas9 and dCas9 are sensitive to nucleosome phasing [124].

There are multiple potential applications of CRISPRi and CRISPRa technology in virology research. CRISPRa offers the ability to quickly and effectively preform a screen for the effect of gene overexpression on a virology endpoint, such as viral fitness or host cell entry. CRISPRa offers advantages to a cDNA expression screen: sgRNAs are smaller than cDNAs, and therefore sgRNA libraries are more easily and cheaply constructed than cDNA libraries; sgRNAs are more readily delivered to target cells than cDNAs, particularly for large genes; sgRNAs can upregulate the expression of gene isoforms; combinations of genes can be more easily induced by multiple sgRNAs.

The first virology-focused CRISPRa genetic screen was recently published. Heaton et al. used CRISPRa to screen for host inhibitory factors that protect cells against influenza virus (IAV) infection [125]. To this end, the authors employed the dCAS9–VP64 fusion variant in conjunction with MS2–p65–HSF1. In this system, the VP64 activator (four copies of VP16) is fused to dCas9, and the transcriptional activity is further increased by appending an sgRNA that binds the bacteriophage MS2 coat protein fused to the NF-κB subunit p65 and to heat shock factor 1 (HSF1). The p65 and HSF1 “Synergistic Activation Mediator” (SAM) is recruited to MS2 RNA hairpins present on the sgRNA [115]. This system was used in a human lung epithelial A549 cell line that stably expresses a fluorescent ZsGreen Cre recombinase reporter. To conduct a CRISPRa-pooled screen, an A549 cell sgRNA library was made by lentivirus transduction and was challenged with Cre-expressing IAV. Specifically, 2 × 10^8^ cells were transduced at a MOI of 0.3 with a lentivirus promoter-targeting sgRNA library. The transduced cells were puromycin-selected, and 72 hours later were exposed to IAV at a MOI of 5.

To identify potential host restriction factors, the authors screened for sgRNAs that blocked IAV Cre-induced ZsGreen expression. The authors used florescence activated cell sorting (FACS) to isolate cells with no ZsGreen signal, suggesting that they were refractory to either IAV uptake or at an early stage of IAV infection, prior to Cre expression. Three biological replicates were performed. sgRNA abundance in input versus ZsGreen negative-sorted cells were measured by deep sequencing of PCR amplicons. The major screen hit was the host glycosyltransferase Beta-1,4-*N*-Acetyl-Galactosaminyltransferase 2 (B4GALNT2), which was identified as being inhibitory for IAV entry. The avian IAV strain used in the screen, like other avian IAV, uses an α2,3-linked sialic acid receptor for entry. By contrast, most human influenza virus strains not adapted to grow in chicken cells, use instead an α2,6-linked sialic acid receptor [126,127]. B4GALNT2 modifies glycans containing α2,3-linked sialic acids, adding a GalNAc moiety to the subterminal galactose of α2,3-linked sialic acid-containing glycans. This modification was found to abrogate avian IAV binding to the host cell surface. Interestingly, B4GALNT2 overexpression restricted infection by all tested avian IAV strains, revealing a pan-avian influenza virus inhibitory host factor and highlighting the power of CRISPRa to study virus–host interactions. B4GALNT2 overexpression caused a greater than 100-fold reduction in IAV infection.

While quite nicely executed, it is notable that the IAV CRISPRa screen had just one strong hit. As noted by the authors, false negatives may have resulted from insufficient levels of target gene upregulation. For instance, the known restriction factors Interferon Induced Transmembrane Protein 3 (IFITM3) and MxA may not have reached sufficient levels to block IAV. In addition, the binary nature of the screen output, in which even low levels of Cre recombinase might have been sufficient to induce ZsGreen expression, may have contributed to the low hit rate.

Though not yet performed, there is ample potential for CRISPRi application to study virus–host interactions, including in areas that are not conductive to study through traditional CRISPR–Cas9 or siRNA screens. For example, long noncoding RNAs (lncRNA) are important for a variety of cellular processes [128,129,130,131]. CRISPR–Cas9 screens of lncRNA targets are technically difficult, since lncRNAs function at the RNA level, and NHEJ-induced indels are often insufficient to block lncRNA function. Likewise, RNAi screens of lncRNA function can be challenging, for example because of off-target effects [132,133] or variable knockdown efficiencies of these nuclear targets [134].

CRISPRi is an appealing strategy to potentially overcome these technical limitations. Liu et al. conducted a CRISPRi screen to investigate lncRNAs that affect cell growth [135]. dCas9–KRAB [114,116,117] was stably expressed in a variety of cell types, and these cells were subsequently transduced with a lentiviral library containing sgRNAs against lncRNA loci. Deep sequencing was then used to measure sgRNA enrichment in passaged versus input cell populations. Interestingly, the effects of CRISPRi-driven lncRNA knockdown was markedly different across the cell types examined. The majority of lncRNA hits affected growth of only a single cell type.

**Figure 6:**
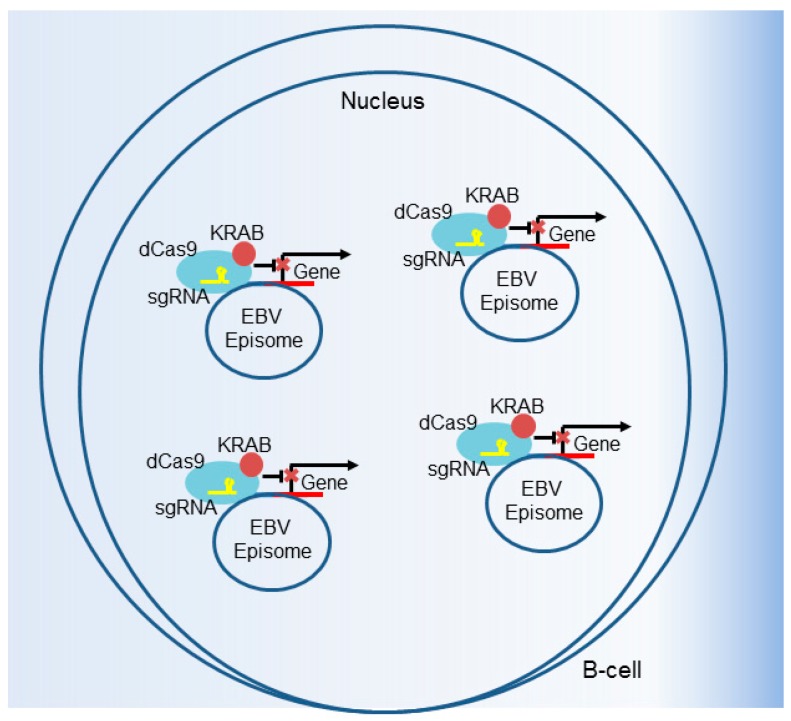
CRISPRi suppression of viral gene expression. CRISPRi can be used to target viral genomes, including multicopy EBV episomes. In this schematic, a sgRNA targets the dCas9–KRAB fusion to an EBV gene to downmodulate its expression. CRISPRi is advantageous when there are multiple copies of a host or viral gene, such as with EBV latently infected B cells which contain 5–50 viral genomes per cell. The targeted disruption with traditional CRISPR–Cas9 of high copy number genes, such as in EBV-infected cells or in areas of tumor cell genome amplification, is difficult because NHEJ repairs a subset correctly and because G2 arrest can also result (see text).

CRISPRi also has the potential to be useful in loss-of-function studies of loci amplified in cancer cell genomes or that are present in multiple copies on viral genomes in infected cells. CRISPR–Cas9 cleavage of regions amplified by copy number gain results in G2 cell cycle arrest, in a manner that correlates with the target number [136]. This gene-independent effect, which even occurs at noncoding sites, can confound the analysis of these polyploid regions by traditional CRISPR–Cas9 gene editing. Another potential technical limitation of traditional CRISPR occurs when targeting viral genomes present at high copy number in a host cell, such as LCLs, which contain ~10–50 viral episomes [137,138] (Figure 6). While not yet associated with G2 arrest, it is unlikely that traditional CRISPR–Cas9 targeting by a single sgRNA will functionally knockout the target gene on every episome. At a low but detectable rate, NHEJ repairs dsDNA breaks in a manner that restores the reading frame. In these settings, CRISPRi is an appealing way to perform loss-of-function studies, as it is less sensitive to copy number amplification and does not generate a DNA damage-driven G2 arrest (Figure 6).

**Figure 7:**
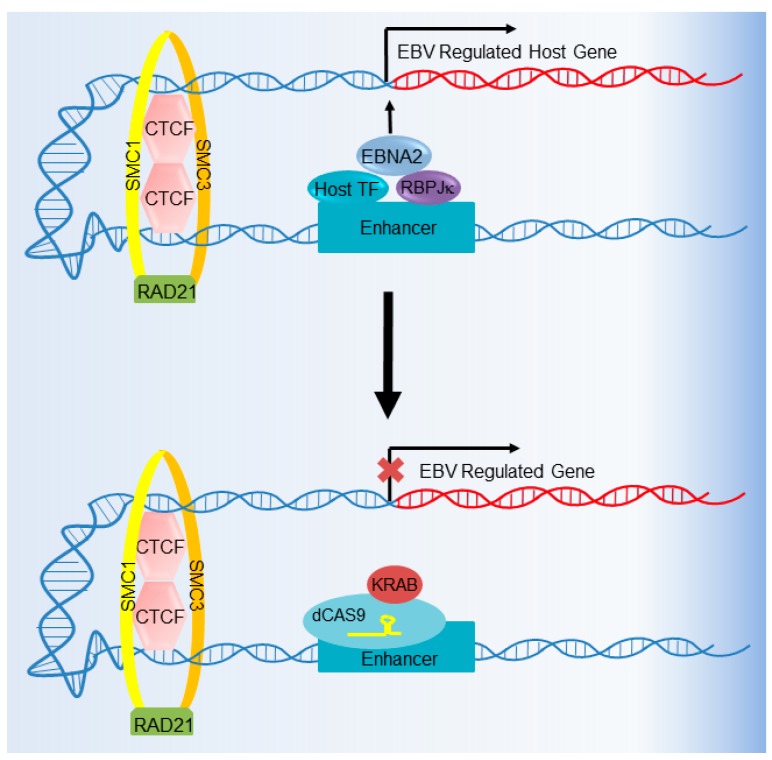
CRISPRi-mediated host enhancer deactivation. CRISPRi can be used to decommission transcription regulatory elements, including host enhancers bound by viral and/or host transcription factors. In this example, CRISPRi is used to deactivate a host enhancer occupied by Recombination Signal Binding Protein for Immunoglobulin κ J Region (RPB-Jκ), Epstein–Barr nuclear antigen 2 (EBNA2), and host transcription factors (TFs). Also shown are the DNA-looping factors CCCTC-Binding Factor (CTCF), Structural Maintenance of Chromosomes 1 (SMC1), SMC3, and RAD21, which facilitate long-range enhancer–promoter interactions.

CRISPRi can also be used to interrogate the importance of host cell enhancers in virus-infected cells. For instance, Chromatin Immunoprecipitation followed by Deep Sequencing (ChIP-seq) identified thousands of host genome binding sites for Epstein–Barr nuclear antigen (EBNA) transcription factors, many of which loop to target gene promoters from large distances away [97,98,105,139] (Figure 7). The biological significance of the vast majority of these EBNA-bound enhancers on target gene expression and B cell growth or survival remains to be tested. We recently used CRISPRi to interrogate the effect of EBNA-bound LCL enhancer deactivation in a proof-of-principle study [140]. Furthermore, multiple EBNA-bound enhancers often loop to a target gene promoter, and these can in theory be simultaneously targeted by multiplex sgRNA approaches, in which the expression of multiple sgRNAs enables the simultaneous deactivation of multiple enhancers [141]. CRISPRi therefore provides a rapid way to interrogate the importance of a viral protein-bound enhancer on host or viral phenotypes. By contrast, the efficiency of generating biallelic enhancer knockouts by traditional Cas9 DNA targeting is low.

## 7. Future Directions

Ongoing technical advances in CRISPR–Cas system development will increasingly support genetic analyses of host–pathogen interactions. Recently, Najm, Doench, and colleagues used CRISPR–Cas9 systems to perform elegant combinatorial knockout screens [12]. In this study, orthogonal Cas9 enzymes from *S. aureus* and *S. pyogenes* were used together to achieve the simultaneous knockout of two host genes. This so-called “Big Papi” (paired *aureus* and *pyogenes* for interactions) approach was used to overcome several technical challenges. These included the reduction in screen efficiency from high recombination frequencies between repetitive lentiviral vector elements, such as when multiple U6 promoters are present in a single vector to drive the expression of two sgRNAs, or from sgRNA competition for Cas9 loading [12]. The Big Papi approach cleverly uses U6 and H1 promoters to express independent sgRNAs, which then program either *S. aureus* or *S. pyogenes* Cas9. In pilot studies, a dual knockout was observed in 50–87% of cells, and the empirically calculated true positive and false-negative rates were encouraging. As a further proof-of-concept, Big Papi was then used to test synthetic lethal and buffering gene-pair interactions in several well-characterized pathways. Focused screens using either double knockout or knockout with CRISPRa found interactions within mitogen activated kinase (MAPK), DNA damage repair, and apoptosis pathways [12].

Big Papi can be performed in a wide range of cells, including those currently used for Cas9 screens. It can therefore, in principle, be used for studies with a wide range of viruses. Combinatorial approaches can potentially overcome the sources of false-negative results, such as when the redundancy between two gene products precludes the identification of a phenotype by single-gene perturbation approaches. Pairwise knockout strategies enhanced the identification of systems-level genetic networks in yeast [142] and can now in principle be done in virus-infected mammalian cells. Intriguingly, Big Papi technology could also be used to study instances where positive and negative signal impact a virological outcome, such as the reactivation from latency. As an example, chemical cocktails are used to induce HIV or EBV reactivation in lymphocytes, typically combining histone deacetylase (HDAC) or DNA methyltransferase inhibitors with a protein kinase C activator. A drawback of this approach is that these compounds target multiple host enzymes and result in cytotoxicity. To systematically analyze optimal targets within these enzyme classes, combinatorial activation and knockout screens could be performed, for instance for optimal HDAC and kinase pairs. Alternatively, since redundancy may limit the phenotypes, CRISPR could be used to knockout pairs of HDAC or DNA methyltranseferase targets in the presence of a protein kinase C (PKC) agonist.

There are, however, several technical Big Papi-screen drawbacks to consider. First, as the name Big Papi implies, the combinatorial approach squares the number of perturbations to be screened. For instance, testing 20 pairwise gene knockouts, using just one sgRNA per gene, requires 400 combinations. With two sgRNAs per gene, 1600 combinations are required. This scale can be problematic for low-throughput screens, such as with flow cytometry-based selection. Whereas multiple sgRNA libraries are now available for traditional CRISPR screens, libraries for combinatorial screens are not yet available and would therefore need to be custom-made for each study. While the pPapi vector is freely available through Addgene (plasmid #96921), relatively large ~140 base pair oligonucleotide inserts are required, adding to the cost of library construction [12].

The CRISPR toolkit continues to expand with the identification of additional CRISPR–Cas systems [143]. CRISPR–Cas13 enables RNA editing and can be engineered to knockdown or even to edit specific RNA messages [144,145]. As an example, Cas13a is an RNA-guided RNA ribonuclease that can be used to manipulate RNA abundance [146]. CRISPR–Cas13 could in principle be used to simultaneously screen host and RNA virus genomes using pooled screen approaches. Though, to our knowledge, it has not yet been used in virology screens, Cas13-based reporter systems have been cleverly used for a rapid and highly sensitive detection of flavivirus genomes [147]. Since the off-target profiles of Cas9 and Cas13 are very likely to be distinct, Cas13 analyses could also be used in validation studies of Cas9 screens, where concordant phenotypes would suggest on-target effects. These and other technical advances will increasingly enable the discovery of how viruses exploit host factors and promise to highlight areas for therapeutic development.
